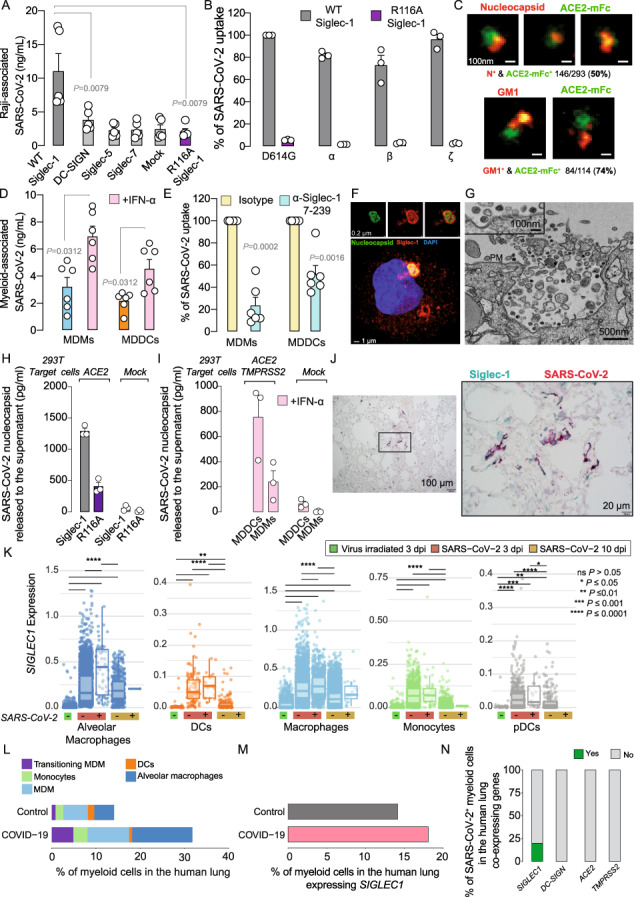# Author Correction to: SARS-CoV-2 interaction with Siglec-1 mediates *trans*-infection by dendritic cells

**DOI:** 10.1038/s41423-022-00893-y

**Published:** 2022-07-11

**Authors:** Daniel Perez-Zsolt, Jordana Muñoz-Basagoiti, Jordi Rodon, Marc Elosua-Bayes, Dàlia Raïch-Regué, Cristina Risco, Martin Sachse, Maria Pino, Sanjeev Gumber, Mirko Paiardini, Jakub Chojnacki, Itziar Erkizia, Xabier Muñiz-Trabudua, Ester Ballana, Eva Riveira-Muñoz, Marc Noguera-Julian, Roger Paredes, Benjamin Trinité, Ferran Tarrés-Freixas, Ignacio Blanco, Victor Guallar, Jorge Carrillo, Julià Blanco, Amalio Telenti, Holger Heyn, Joaquim Segalés, Bonaventura Clotet, Javier Martinez-Picado, Júlia Vergara-Alert, Nuria Izquierdo-Useros

**Affiliations:** 1 IrsiCaixa AIDS Research Institute, 08916 Badalona, Spain; 2 IRTA, Centre de Recerca en Sanitat Animal (CReSA, IRTA-UAB), Campus de la UAB, 08193 Bellaterra (Cerdanyola del Vallès), Spain; 3 CNAG-CRG, Centre for Genomic Regulation (CRG), Barcelona Institute of Science and Technology (BIST), 08028 Barcelona, Spain; 4 Centro Nacional de Biotecnología, CSIC, 28049 Madrid, Spain; 5 Division of Microbiology and Immunology, Yerkes National Primate Research Center, Emory University, Atlanta, GA USA; 6 Department of Pathology and Laboratory Medicine, School of Medicine, Emory University, Atlanta, GA USA; 7 Division of Pathology, Yerkes National Primate Research Center, Emory University, Atlanta, GA USA; 8 Germans Trias i Pujol Research Institute (IGTP), Can Ruti Campus, 08916 Badalona, Spain; 9 Barcelona Supercomputing Center (BSC), 08034 Barcelona, Spain; 10 Catalan Institution for Research and Advanced Studies (ICREA), 08010 Barcelona, Spain; 11 Present Address: University of Vic—Central University of Catalonia (UVic-UCC), Vic, 08500 Spain; 12 Department of Integrative Structural and Computational Biology, Scripps Research, La Jolla, CA 92037 USA; 13 Universitat Pompeu Fabra (UPF), Barcelona, Spain; 14 UAB, CReSA (IRTA-UAB), Campus de la UAB, 08193 Bellaterra (Cerdanyola del Vallès), Spain; 15 Departament de Sanitat i Anatomia Animals, Facultat de Veterinària, UAB, 08193 Bellaterra (Cerdanyola del Vallès), Spain

**Keywords:** Infectious diseases, Mechanisms of disease

Correction to: *Cellular & Molecular Immunology* 10.1038/s41423-021-00794-6, published online 15 November 2021

In the version of this correspondence initially published, one of the SARS-CoV-2 variants used in Fig. 1B, which was originally described in the article as the SARS-CoV-2 variant ‘B.1.1.248.2 Gamma’, is actually the ‘P.2 Zeta’ SARS-CoV-2 variant of interest. The GISAID accession ID EPI_ISL_1831696 provided is correct, but it belongs to the Zeta variant. The results and conclusions are not affected by this unintentional inaccuracy.